# Mechanism of Irrigation Before Low-Temperature Exposure on Mitigating the Reduction in Yield Loss and Spikelet Abortion at the Jointing Stage of Wheat

**DOI:** 10.3390/antiox13121451

**Published:** 2024-11-26

**Authors:** Yangyang Wang, Mao Wang, Peipei Tian, Dechao Ren, Haiyan Zhang, Geng Ma, Jianzhao Duan, Chenyang Wang, Wei Feng

**Affiliations:** 1 College of Resources and Environment, Henan Agricultural University, Zhengzhou 450002, China; 5liebely3@stu.henau.edu.cn; 2 College of Agronomy, State Key Laboratory of Wheat and Maize Crop Science, Henan Agricultural University, Zhengzhou 450002, China; 3 National Engineering Research Center for Wheat, Zhengzhou 450002, China; 4 Postdoctoral Station of Crop Science, Henan Agricultural University, Zhengzhou 450002, China; 5 Wheat Research Laboratory, Shangqiu Academy of Agriculture and Forestry Sciences, Shangqiu 476000, China

**Keywords:** low temperature, irrigation before low-temperature exposure, fertility rate of different spike positions, physiological mechanisms, antioxidant capacity

## Abstract

The increasing frequency of low-temperature events in spring, driven by climate change, poses a serious threat to wheat production in Northern China. Understanding how low-temperature stress affects wheat yield and its components under varying moisture conditions, and exploring the role of irrigation before exposure to low temperatures, is crucial for food security and mitigating agricultural losses. In this study, four wheat cultivars—semi-spring (YZ4110, LK198) and semi-winter (ZM366, FDC21)—were tested across two years under different conditions of soil moisture (irrigation before low-temperature exposure (IBLT) and non-irrigation (NI)) and low temperatures (−2 °C, −4 °C, −6 °C, −8 °C, and −10 °C). The IBLT treatment effectively reduced leaf wilt, stem breakage, and spikelet desiccation. Low-temperature stress adversely impacted the yield per plant—including both original and regenerated yields—and yield components across all wheat varieties. Furthermore, a negative correlation was found between regenerated and original yields. Semi-spring varieties showed greater yield reduction than semi-winter varieties, with a more pronounced impact under NI compared to IBLT. This suggests that the compensatory regenerative yield is more significant in semi-spring varieties and under NI conditions. As low-temperature stress intensified, the primary determinant of yield loss shifted from grain number per spike (GNPS) to spike number per plant (SNPP) beyond a specific temperature threshold. Under NI, this threshold was −6 °C, while it was −8 °C under IBLT. Low-temperature stress led to variability in fruiting rate across different spike positions, with semi-spring varieties and NI conditions showing the most substantial reductions. Sensitivity to low temperatures varied across spikelet positions: Apical spikelets were the most sensitive, followed by basal, while central spikelets showed the largest reduction in grain number as stress levels increased, significantly contributing to reduced overall grain yield. Irrigation, variety, and low temperature had variable impacts on physiological indices in wheat. Structural equation modeling (SEM) analysis revealed that irrigation significantly enhanced wheat’s response to cold tolerance indicators—such as superoxide dismutase (SOD), proline (Pro), and peroxidase (POD)—while reducing malondialdehyde (MDA) levels. Irrigation also improved photosynthesis (Pn), chlorophyll fluorescence (Fv/Fm), and leaf water content (LWC), thereby mitigating the adverse effects of low-temperature stress and supporting grain development in the central spike positions. In summary, IBLT effectively mitigates yield losses due to low-temperature freeze injuries, with distinct yield component contributions under varying stress conditions. Furthermore, this study clarifies the spatial distribution of grain responses across different spike positions under low temperatures, providing insights into the physiological mechanisms by which irrigation mitigates grain loss. These findings provide a theoretical and scientific basis for effective agricultural practices to counter spring freeze damage and predict wheat yield under low-temperature stress.

## 1. Introduction

With the rapid growth in global population and food demand, it is essential to increase global crop production by 60% by mid-century to meet future needs [1]. Wheat, one of the most extensively cultivated crops worldwide, is fundamental to maintaining food security [2]. However, fluctuations in the global climate significantly increased the frequency of low-temperature events during spring, contributing to instability in wheat production cycles and severely affecting wheat growth and development [3]. In recent years, low-temperature stress has caused significant losses in wheat production across several regions, including China [4], the United States [5], Australia [6], and parts of Europe [7,8]. This emerging threat poses serious risks to wheat food security. In China, particularly within the Yellow–Huai River Wheat Region—a major wheat-producing area—low-temperature stress frequently occurs from the jointing to the booting stages of wheat development. During this critical transition from vegetative to reproductive growth, a notable decline in the frost resistance of wheat occurs, increasing the risk of yield losses. When canopy temperatures plummet to 0 °C or below, extreme cold can induce frost damage, substantially impacting wheat production with yield losses ranging from 14% to 85% [9,10,11].

The jointing stage is a critical phase in wheat growth and development, particularly susceptible to low-temperature stress, which results in substantial yield reductions by affecting spike numbers, grains per spike, aboveground biomass, and photosynthetic capacity [8,12,13,14]. During early spike differentiation, although the flag leaf sheath partially protects the developing spikelet, the spikelet remains highly delicate and moisture-rich, making it sensitive to environmental factors such as temperature and soil moisture [15]. Sub-freezing temperatures can trigger freezing damage that disrupts typical spikelet and floret development, resulting in higher rates of floret abortion, increased numbers of infertile florets, and impairment of spikelet cellular structure. Such disruptions contribute to spikelet dehydration, diminished floret differentiation, and reduced grain count, ultimately increasing spikelet mortality and significantly reducing overall yield [16,17,18]. In a study by Li et al. [4], low-temperature exposure during the jointing stage caused a notable 6% to 13% decrease in wheat spike numbers. Fuller et al. [16] reported grain losses ranging from 10% to 100% during the vegetative stage when temperatures dropped between −5 °C and −13 °C, primarily due to damage to flag leaves and young spikes. Severe low-temperature stress could even lead to primary tiller death, which, in some cases, stimulates tiller regrowth, into spikes, compensating partially for yield losses. Wu et al. [19] found decreased primary wheat spikes during the jointing stage when exposed to low-temperature treatments (−1 °C to −9 °C), with an increase in regenerated spikes sometimes surpassing primary spike yield. Liu et al. [20] noted that exposure to −6 °C led to the death of primary tillers but resulted in rapid tiller regrowth and increased seed yields compared to non-exposed plants. Wheat yield, determined by spike number, grains per spike, and 1000-grain weight, shows a notable decrease in response to low-temperature stress at the jointing stage, with the severity of the reduction positively correlating to the intensity of stress. Ji et al. [21] observed yield reductions in winter wheat ranging from 3.1% to 56.4% under varying low-temperature conditions, with spike numbers and grains per spike being particularly sensitive. A pot experiment by Liu et al. [20] highlighted low-temperature induced grain yield reductions mainly attributable to decreased spike and grain numbers. Notably, apical spikelets exhibited the highest sensitivity to low temperatures, followed by basal and central spikelets. Lin et al. [22] observed that low temperatures delayed plant growth and inhibited spike and floret differentiation, resulting in severe damage to both apical and basal spikelets, while the glumes of the central spikelets remain relatively well developed. Similarly, Zhang et al. [23] reported that low-temperature stress during anther differentiation negatively affected photosynthetic activity, which is crucial for dry matter accumulation in wheat, thereby slowing the growth of young spikes and ultimately reducing grain yield.

Low-temperature stress damages the cell membrane, triggering a cascade of physiological and biochemical responses to mitigate the damage, including the activation of antioxidant and osmoregulatory systems [24]. During low-temperature exposure, the significant accumulation of reactive oxygen species (ROS) prompts increased activity of wheat’s antioxidant enzymes, such as superoxide dismutase (SOD) and peroxidase (POD). This activity helps maintain ROS homeostasis, reduce membrane lipid peroxidation, and subsequently decrease malondialdehyde (MDA) levels, a byproduct of lipid peroxidation [25,26,27]. Concurrently, the osmoregulatory system maintains cellular structural homeostasis by elevating levels of osmoregulatory substances such as proline (Pro) [28]. Photosynthesis, a highly sensitive physiological process in plants, responds to low temperatures by accumulating ROS and causing ester peroxidation damage to chloroplasts and cell membrane structures, resulting in the destruction of chloroplast structures, severe damage to the photosynthetic apparatus, and reduced photosynthetic efficiency [29,30,31]. Chlorophyll fluorescence, a sensitive tool for detecting plant responses to environmental stress, has been widely employed to analyze the impact of low temperatures on leaf photosynthesis using various chlorophyll fluorescence parameters [32,33]. Specifically, the maximum quantum yield (Fv/Fm) serves as a critical parameter for rapidly and non-destructively assessing frost damage in crops exposed to low temperatures. A decrease in Fv/Fm is typically associated with damage to the PSII reaction center and is closely linked to the severity of low-temperature stress [34]. Li et al. [4] found that low temperatures (8 °C below ambient levels) during the jointing stage significantly reduced the gas exchange rate and the maximum quantum efficiency of photosystem II in wheat, ultimately resulting in a 5% to 14% decrease in grain yield. Xu et al. [35] demonstrated that exposure of wheat to low temperatures of 4 °C and −4 °C during the anther differentiation period caused the net photosynthetic rate (Pn) of flag leaves to decrease by 26.8% and 42.2%, respectively, while the yield per plant decreased by 24.1% and 48.2%. A four-hour exposure to −4 °C led to an initial increase followed by a decrease in antioxidant enzyme activities (SOD, POD, and CAT) within eight days, with malondialdehyde (MDA) content consistently higher than that in the control group [28]. Ejaz et al. [36] found that chlorophyll content, antioxidant enzyme activities and hydrogen peroxide (H_2_O_2_) interacted under low-temperature stress.

The impact of low-temperature stress on wheat is influenced by various factors, encompassing cultivar type, temperature levels, stress duration, as well as soil moisture content, and water and fertilizer management strategies. Notably, frost damage is associated with soil moisture levels, and maintaining adequate soil moisture is effective in alleviating the detrimental effects of low temperatures on wheat yield [37,38]. Meng et al. [37] demonstrated that when soil moisture content fell below 10%, a notable decrease in spike number, grains per spike, and yield per plant occurred compared with treatments using medium (10–20%) and wet (>20%) soil moisture conditions. However, the efficacy of irrigation before low-temperature exposure (IBLT) as a management strategy to alleviate yield losses due to low temperatures has received relatively limited research attention. Therefore, investigating the mitigating effect of irrigation on low-temperature stress and elucidating the specific mechanisms of low-temperature stress on wheat are of substantial production value for mitigating low-temperature damage.

In summary, previous studies have primarily focused on the degree and duration of low temperatures affecting the physiological characteristics and yield of wheat. However, practical wheat production involves a complex interplay of various cultural and environmental factors. There is a lack of studies examining the impact of low-temperature stress on wheat’s physiological characteristics and yield components under multifaceted conditions, particularly research focusing on the compensatory effects of regenerating spikes and the spatial variation of spike positions. To address this, the current study simulated spring freezing damage using a low-temperature simulation chamber under two irrigation conditions: irrigation before low-temperature exposure (IBLT) and non-irrigation (NI). Additionally, representative wheat varieties with varying vernalization types that are extensively cultivated were selected. The purpose of this study is to clarify wheat yield and its components’ response to low temperatures under different irrigation conditions and evaluate the contribution of these factors to the overall yield. We also evaluated the sensitivity of the spatial distribution of wheat spike positions to low temperatures and examined the response of physiological characteristics to low temperatures under various moisture conditions. By establishing correlations between physiological characteristics and grain development at different spike positions, this study aims to explore the potential of IBLT in mitigating frost damage effects on wheat yield development. Furthermore, we aim to provide a theoretical foundation and technical guidance for enhancing wheat production resilience against frost damage.

## 2. Materials and Methods

### 2.1. Experimental Design and Treatments

The experiments were conducted during the 2018–2020 growing seasons at the experimental Demonstration Centre of the Academy of Agricultural and Forestry Sciences, Shangqiu City, Henan Province, China (115°72′ E, 34°54′ N). The studied wheat cultivars included semi-spring varieties (Yanzhan 4110 (YZ4110) and Lankao 198 (LK198)) and semi-winter varieties (Zhengmai 366 (ZM366) and Fengde Cunmai 21 (FDC21)) (Table 1), sown in cylindrical hollow tubes (height and interior diameter were 35 cm and 25 cm, respectively), with a planting density of 11 plants per pot. Seeds were sown on 9 November 2018 and 12 November 2019 for both cultivars. The top of the plot remained open while a wire mesh was fixed to the bottom. Before sowing, all pots were buried in the ground, and the top was made level with the soil surface, ensuring a growth environment equivalent to the field. Soil for the experiment was sieved, dried, and weighed before use. The soil’s fertility averaged 13.4 g·kg^−1^ organic matter, 74.2 mg·g^−1^ hydrolyzed nitrogen, 36.31 mg·g^−1^ available phosphorus, and 129.48 mg·g^−1^ available potassium. Each variety was planted in 192 pots, half of which were used for irrigation treatments, and the remaining half were left without watering. Additional management practices, including irrigation, pest, and disease control, adhered to local wheat cultivation standards, ensuring that moisture, pests, or weeds did not hinder wheat growth. Once the wheat reached the jointing stage (Zadoks 31) [39], two moisture treatments were applied to each variety: non-irrigation (NI) with 13~15% soil moisture and irrigation before low-temperature exposure (IBLT) with 20~22% soil moisture. The soil moisture was determined by collecting soil at 5 cm depth in each pot and calculated using the drying method. The irrigation amount was then determined based on this value and the pot volumes, ensuring that each pot maintained the soil moisture content within the desired range. The potted plants were relocated to the greenhouse for low-temperature treatment 5 days after irrigation.

The low-temperature simulation room measured 7 m in length, 5 m in width, and 2.6 m in height. At the top of the room, two nylon cloth ventilation tunnels were connected to two refrigeration compressors outside the room. For each tunnel, several circular holes with an interval of 20 cm were drilled on both sides, serving as ventilation outlets. The hole’s diameter was 5 cm. When the experiment began, cold air passed through the holes and formed a sub-freezing temperature space at the top of a horizontal surface with a height of 1.9 m. The cold air sank evenly to simulate a temperature decrease process similar to that which occurs naturally in a field. Five temperature levels (T1: −2 °C, T2: −4 °C, T3: −6 °C, T4: −8 °C, T5: −10 °C) were employed alongside a control (CK) using the same wheat cultivar but without low-temperature treatment. Notably, the CK was not exposed to natural frost damage on the treatment day. The low-temperature stress duration was set to 8 h. Initially, a gradual temperature decrease occurred over the first 2 h, followed by maintaining the minimum temperature for 5 h, and in the final hour, the temperature was restored to ambient conditions. Following the low-temperature stress, the potted plants were transferred from the simulation room back to the field to resume their original growth conditions. Physiological indices were then measured three days post-stress.

### 2.2. Sampling and Measurements

#### 2.2.1. Observation of Wheat Plant and Floret Morphological Damage

Three days following the conclusion of the low-temperature treatment, we observed and photographed the morphological damage in wheat plants, specifically focusing on the middle section of young spikelets, the spikelets at the 9th position, and the floret primordia at the 1st position on the spikelets.

#### 2.2.2. Leaf Water Content, Photosynthetic Rate, and Chlorophyll Fluorescence Parameters (Fv/Fm)

The leaf water content (LWC) of wheat leaves from each treatment was determined by measuring their dry weight and fresh weight and calculating it according to Formula (1). Three plants were selected at random in each pot with three replicates.

(1) LWC=(Fw−Dw)/Fw×100

where Dw is the dry weight of the leaf, and Fw is the fresh weight of the leaf.

Photosynthetic measurements were conducted from 9:00 to 12:00 Beijing time under clear skies, using the LI-COR 6400 portable photosynthesis system (Lincoln, NE, USA). We utilized leaf chambers with red and blue light sources, setting the photosynthetically active radiation (PAR) at 1100 μmol·m^−2^ s^−1^ to assess the net photosynthetic rate (Pn) of the topmost fully expanded leaf, three leaves were tested for each treatment.

#### 2.2.3. Leaf SOD, POD, and MDA

For each treatment, one pot was selected, and three wheat plants exhibiting uniform growth were selected. The topmost fully expanded leaf from each plant was harvested, wrapped in tin foil for preservation, and immersed in liquid nitrogen. All collected samples were stored at −80 °C for subsequent enzyme activity analysis, specifically for SOD, POD, and MDA. Leaf samples were transferred into a mortar, doused with liquid nitrogen, and ground using a pestle. Subsequently, SOD and POD activities were assessed using the nitroblue tetrazolium (NBT) method and visible spectrophotometry, while the MDA content was determined using the thiobarbituric acid (TBA) method [40,41]. Antioxidant enzyme and MDA levels were analyzed using micro-methods (zymography) with SOD, POD, and MDA kits from Suzhou Keming Biotechnology Co. Ltd, Suzhou, China, following the manufacturer’s guidelines. The specific procedure involved homogenizing 0.1 g of leaf tissue with 1 mL of extraction buffer, followed by ice bath homogenization and centrifugation at 8000× *g* and 4 °C for 10 min. Afterward, the supernatant and reagents were combined in a 96-well plate, and the absorbance was measured using an enzyme counter, with SOD and POD absorbance values determined at 560 nm and 470 nm, respectively. A560(CK) and A560(D) are the absorbance values at 560 nm for the control and test tubes respectively. In addition, record the absorbance values at 470 nm after 1 min as A1 and after 2 min as A2. The absorbance of MDA was measured at 532 nm and 600 nm, denoted as A532 and A600, respectively. The activities of antioxidant enzymes (SOD, POD) (2) and (3) and the extent of membrane lipid peroxidation (MDA) (4) were calculated using the manufacturer’s formula. SOD activity, POD activity and MDA content were determined in three biological replicates of each treatment.

(2) $$SOD\;Activity\;(U/g\;Fresh\;Weight)=\frac{11.11\times (\frac{A560\left(CK\right)-A560\left(D\right)}{A560\left(CK\right)}\times 100\%)}{(1-(\frac{A560\left(CK\right)-A560\left(D\right)}{A560\left(CK\right)}\times 100\%))\times W}$$

(3) $$POD\;Activity\;(U/g\;Fresh\;Weight)=\frac{4000\times (A2-A1)}{W}$$

(4) $$MDA\;Content\;(nmol/g\;fresh\;weight)=\frac{51.6\times (A532-A600)}{W}$$

*W*: is the mass of the sample (g).

#### 2.2.4. Leaf Proline Content

The Pro content was quantified using the sulfosalicylic acid method [42]. This involved weighing 0.5 g of the flag leaf, three biological replicates for each treatment, adding 5 mL of 3% sulfosalicylic acid, boiling for 10 min, then centrifuging to obtain 2 mL of the supernatant. To this solution, 2 mL each of glacial acetic acid and 2.5% acidic ninhydrin were added, followed by thorough shaking. The mixture was then heated in a boiling water bath for 60 min to allow for color development, cooled to room temperature, and mixed with 5 mL of toluene. The mixture was shaken and left to stand for phase separation. Following phase separation, the toluene layer was collected, and its absorbance was measured at 520 nm. A standard curve was established using different concentrations of Pro to calculate the Pro content. The Pro content was then determined based on this curve and expressed in micrograms per gram (ug/g).

### 2.3. Grain Yield and Yield Component Determination

After the wheat reached maturity, three replications of 3 pots of wheat with 9 pots in total were randomly selected from each treatment to analyze the spike number per plant (SNPP), grain number per spike (GNPS), 1000-grain weight (TGW), grain yield per plant (GYPP) and the spatial distribution of grain number and individual grain weight of wheat under low-temperature conditions. Additionally, for each treatment, 10 spikelets were randomly selected, and based on their vertical positions, the spikelets were categorized into the apical, central, or basal sections. The number of grains and their distribution across these sections were analyzed, followed by calculating the average values.

In the T3–T5 treatments, the primary wheat tillers displayed varying mortality rates when exposed to different moisture conditions. This led to a shift in the plant’s carbon and nitrogen supply from primary tillers to late tillers, roots, and axillary buds. Consequently, this shift facilitated the rapid growth of regenerative tillers, culminating in spike formation and effective yield. Consequently, this study’s analysis of spike count per plant encompassed both primary and regenerated spikes. This study’s criteria for differentiating primary and regenerated tillers were based on Wu et al. [19]. Primary tillers are the main stems that develop normally before the jointing stage, including early-stage tillers. In contrast, regenerated tillers are those formed post-low temperature stress exposure at the jointing stage.

### 2.4. Data Processing Analysis

A multifactorial ANOVA, utilizing SPSS 26.0, analyzed the two-year experimental dataset to assess the impact of low-temperature stress under various watering conditions on wheat yield, yield components, Comparisons were analyzed using Duncan’s multiple range method, and differences were considered statistically significant if the *p*-value was less than 0.05. Additionally, SPSS 26.0 facilitated a multivariate linear regression analysis to explore the relationships between the SNPP, GNPS, and TGW of a single plant under different watering conditions during low-temperature stress.

Graphs representing these data were created using Microsoft Excel 2016 and Origin 2023b. Furthermore, SEMs were constructed using SPSS 26.0 and SPSS Amos 24.0 to analyze how irrigation treatments influenced the physiological mechanisms underlying the mitigation of spike number reduction in wheat under low-temperature stress.

## 3. Results

### 3.1. Effects of Irrigation Before Low-Temperature Exposure on the Development of Leaves and Young Spikes in Wheat

**Figure 1:**
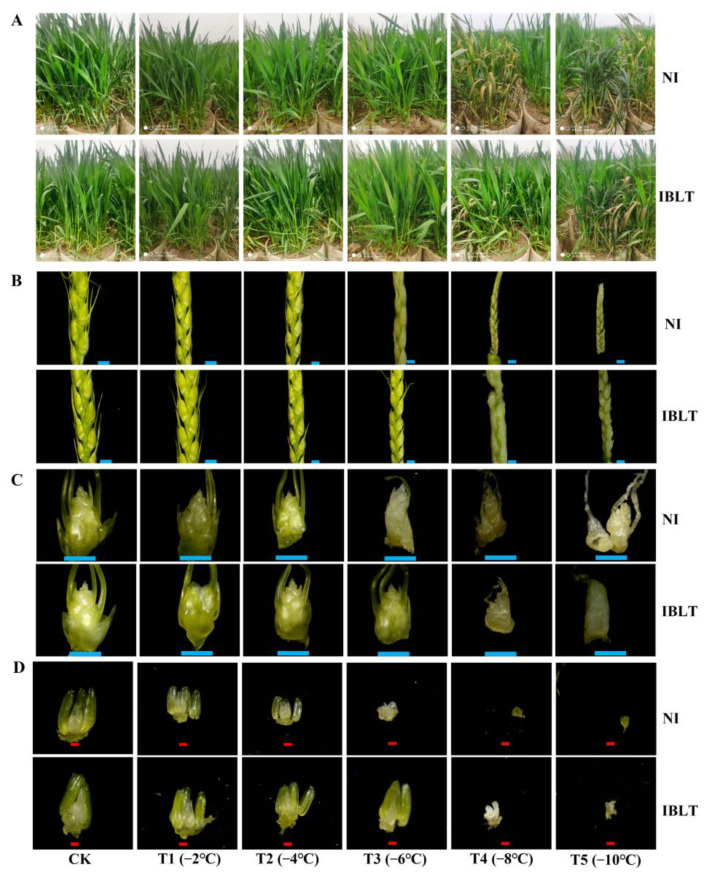
The wheat variety depicted is YZ4110. Panel (**A**) displays the freezing response of YZ4110 plants under two moisture conditions: NI (non-irrigation) and IBLT (irrigation before low-temperature exposure), across different low-temperature levels (CK, −2 °C, −4 °C, −6 °C, −8 °C, and −10 °C). Panel (**B**) shows the effects of irrigation and low-temperature treatments on the development of young spikes. Panel (**C**) details the effects of irrigation and low-temperature treatments on the development of the 9th spikelet of young spikes. Panel (**D**) presents the effects of irrigation and low-temperature treatments on the development of the 1st floret of the 9th spikelet. The ‘Red bar’ in the plots represents 200 μm, while the ‘Blue bar’ represents 1 mm.

As depicted in Figure 1, within the T1–T2 temperature ranges, neither NI nor IBLT treatments induced significant morphological changes in plants or young spikes. However, as the temperature continued to decreased—starting from NI-T2 and IBLT-T3—plants exhibited signs of stress, including drying and yellowing at the leaf margins. With increased low-temperature stress, extensive wilting and leaf drying were observed, accompanied by stem breakage. These findings clearly indicate that frost damage in wheat plants subjected to IBLT treatment was less severe compared to those under NI conditions.

Frost damage in young spikelets is a primary factor leading to reduced wheat production under low-temperature stress. The central spikelet in a young spike undergoes its most rapid development during its differentiation stage; therefore, the ninth spikelet was selected for observation. Figure 1 illustrates that, under identical moisture conditions, the severity of the damage to young spikelets, spikelets, and florets progressively increased as the temperature decreased. Under T3 conditions, wheat spikelets and florets in the NI treatment exhibited dehydration. Under severe low-temperature stress, heavy icing and water loss occurred, resulting in an inability to maintain normal morphology, thereby causing irreversible damage. Conversely, under the IBLT-T3 treatment, wheat spikelets and florets largely maintained their morphological integrity, with less severe dehydration. However, upon reaching T4, dehydration occurred under both NI and IBLT moisture conditions.

### 3.2. Effects of Irrigation Before Low-Temperature Exposure on the Yield of Wheat at the Jointing Stage

**Figure 2:**
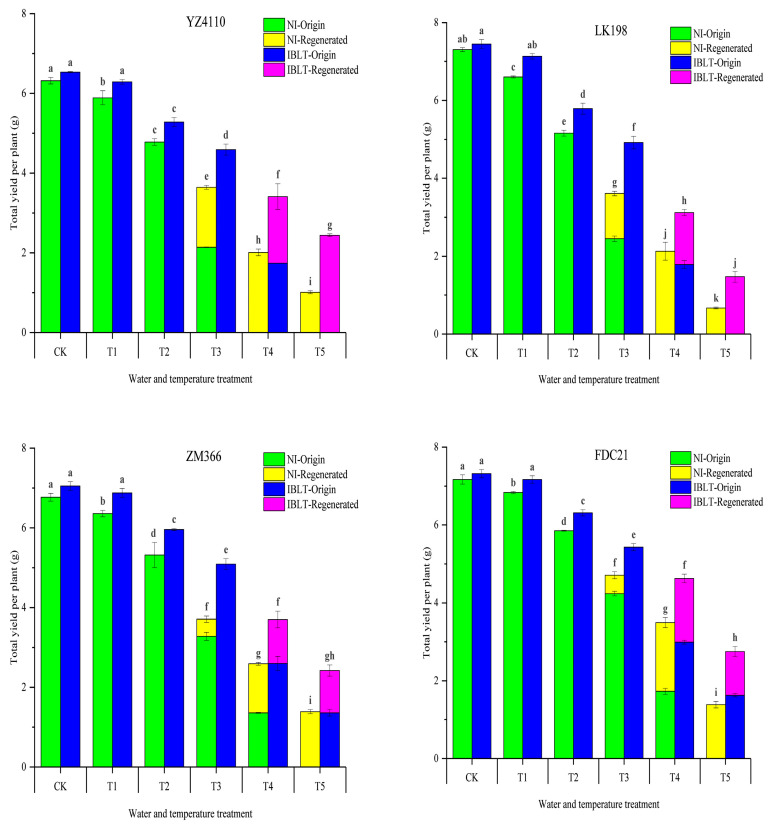
Effects of low-temperature stress treatments (T1–T5) on yield per plant on the original spikes (O) and regenerated spikes (R) in two types of wheat cultivars. Vertical bars represent the standard deviations. Values for the same wheat cultivar type followed by different lowercase letters indicate significant differences at 0.05 level. CK, control; NI, non-irrigated treatments; IBLT, irrigation before low-temperature exposure. Low temperatures for the five treatments were T1: −2 °C, T2: −4 °C, T3: −6 °C, T4: −8 °C, T5: −10 °C. Vertical bars represent the standard deviation of the mean.

As shown in Figure 2, the total yield per plant (O+R) for the different wheat types showed a continuous downward trend as the temperature decreased, and the decline in yield per plant in identical temperature treatments differed with respect to cultivar and water content. When the temperature was between the T1 and T5 treatment temperatures, the mean decline in values for the yields of the semi-spring cultivars (YZ4110 and LK198) and the semi-winter cultivars (ZM366 and FDC21) in the NI condition were 8.2%, 26.84%, 46.48%, 69.51%, 87.42%, and 5.4% (semi-spring) and 19.91%, 39.76%, 56.53%, and 80% (semi-winter), respectively, and the differences in the declines reached a significant level. In the IBLT condition, the difference between the cultivars for the T1 treatment was not significant, and the yield per plant in the semi-spring and semi-winter cultivars decreased by 4.06% and 2.3%, respectively. When the temperature decreased to between T2 and T5, the differences in the yield reductions were significant. The yields per plant in the semi-spring cultivars in treatments T2, T3, T4, and T5 decreased by 20.77%, 31.89%, 53%, and 71.48%, while the yields per plant in the semi-winter cultivars decreased by 14.63%, 26.81%, 42.13%, and 64.05%, respectively. The findings demonstrated that under identical low-temperature conditions, semi-winter varieties exhibited fewer adverse effects and less yield reduction across all treatments compared to semi-spring varieties.

When low-temperature stress intensifies, freezing damage induces the death of part of the original tillers, and the regenerated tillers grow rapidly and restore the yield to a certain degree, which compensates for the original yield. The more severe the stress, the greater the number of regenerated tillers produced. In the NI-T3 and IBLT-T3 treatments, the original yields of the semi-spring cultivars decreased by 66.29% and 31.89%, respectively, compared to the CK, whereas the yields of the semi-winter cultivars were reduced by 46.21% and 26.81%. As temperatures continued to drop, the original yield of the semi-spring cultivars decreased by 74.68% in IBLT-T4, while no yield was obtained in the NI-T4, IBLT-T5, and NI-T5 treatments due to the death of the original spikes. In the NI-T4, IBLT-T4, and IBLT-T5 treatments, the number of original spikes in the semi-winter cultivars decreased by 77.89%, 61.14%, and 79.22%, respectively, while the original spikes in the NI-T5 treatment produced no yield.

Next, we specifically analyzed the yield of the regenerated spikes for each temperature treatment. When the temperature was between T3 and T5, the compensatory effect on yield caused by the regenerated spikes differed depending on the cultivar type, irrigation condition, and temperature. In the NI-T3 treatment, the yields of the regenerated spikes in the semi-spring cultivars and the semi-winter cultivars increased by 19.81% and 6.45%, respectively; when the temperature decreased to T4, the yield of the regenerated spikes in the semi-spring cultivars increased by 30.49% and 21.67% in the NI-T4 and IBLT-T4 treatments, respectively, and the number of regenerated spikes in the semi-winter cultivars increased by 24.36% and 19%, respectively. For the NI-T5 and IBLT-T5 treatments, the compensatory yield effect of the regenerated spikes in the semi-spring cultivars reached 12.58% and 28.52%, and in the semi-winter cultivars it reached 19.96% and 15.17%. When the low temperature exceeds the plant’s bearing capacity, some wheat plants in the NI treatment die, and regenerated tillers are not produced. This results in an overall decrease in the number of regenerated tillers, reducing the compensatory effect of the regenerated spikes on the primary yield in the T5 treatment compared with the T4 treatment for some cultivars.

### 3.3. Effects of Irrigation Before Low-Temperature Exposure on the Yield Components of Wheat at the Jointing Stage

According to Table 2, the yield components (SNPP, GNPS, TWG) progressively declined as the temperature decreased. When the temperature dropped to T1, a significant decrease in yield components was observed in both the semi-spring cultivars (YZ4110 and LK198) and the semi-winter cultivars (ZM366 and FDC21) under the NI condition. In contrast, under IBLT conditions, only the semi-spring cultivars showed a significant decrease in the number of grains per spike.

For the same irrigation conditions, when the temperature decreased to T2, significant reductions in SNPP, GNPS, and TWG were observed across the cultivars. However, under both irrigation conditions (NI and IBLT), no significant differences in TGW were noted for ZM366, whereas significant differences were observed in yield components for the other cultivars. Under NI conditions, the mean reductions in yield components for the semi-spring cultivars (YZ4110 and LK198) were 15.12% (SNPP), 6.71% (TWG), and 9.07% (GNPS). In contrast, under IBLT conditions, SNPP, GNPS, and TWG were reduced by 9.84%, 5.32%, and 7.34%, respectively. For the semi-winter cultivars (ZM366 and FDC21), the yield components in the NI condition were reduced by 8.44% (SNPP), 4.93% (TWG), and 7.7% (GNPS), respectively. In the IBLT condition, they were reduced by 5.31%, 4.06%, and 6.19% compared with the CK.

When the temperature decreased to T3, the yield components between the irrigation and temperature treatments differed significantly. Compared with the CK, the yield components (SNPP, GNPS, TWG) in the semi-spring cultivars in the NI condition were reduced by 46.11%, 10.59%, and 33.66%, respectively, and by 21.37%, 2.8%, and 16.19% in the IBLT condition. Compared with the CK, the yield components in the semi-winter cultivars in the NI-T3 treatment decreased by 23.86%, 7.77%, and 23.47%, respectively, and by 11.41%, 2.33%, and 14.68% in the IBLT-T3 treatment. Among the above results, the TWG increased in the T3 treatment, which is due to a trade-off between the three yield components. The low temperature caused SNPP and GNPS to decrease, but it led to an increase in the TWG. When the temperature decreased to T4, in the NI condition, all the original spikes on the semi-spring cultivars died, and the yield components for the original spikes on the semi-winter cultivars (the average value of the two cultivars) decreased by 62.87%, 3.68%, and 37.24%, respectively, for the SNPP, TWG, and GNPS. In the IBLT condition, the declines in the semi-spring cultivars for the tree yield components were 54.71%, 12.86%, and 35.94%, and the semi-winter cultivars decreased by 48.28%, 8.27%, and 19.0%, respectively. When the temperature decreased to T5, the original spikes on the semi-spring cultivars died, and the declines in SNPP, GNPS, and TWG for the semi-winter cultivars were 64.45%, 13.5%, and 32.26%, respectively.

For the regenerated spikes, the temperature necessary to regenerate spikes in wheat for different cultivars differed between the two irrigation treatments. The more intense the stress, the more regenerated spikes grew. In the T3 treatment, regenerated spikes only grew in the NI condition, and the SNPP on the semi-spring cultivars and semi-winter cultivars increased by 39.48% and 13.09%, respectively. In the T4 treatment, the number of regenerated spikes in the two irrigation conditions had a compensatory effect on the yield to a certain extent, and SNPP on the semi-spring cultivars increased by 73.51% (NI) and 45.04% (IBLT). The SNPP in the semi-winter cultivars increased by 45.14% and 33.87% in NI and IBLT, respectively. When the temperature decreased to T5, the number of regenerated spikes in the semi-spring cultivars in the NI condition was less than in NI-T4 but increased by 41.2% compared with the CK. In comparison, the number of regenerated spikes in the IBLT condition increased by 68.16%. The number of regenerated spikes in the semi-winter cultivars increased by 49.92% and 35.35% under the two irrigation conditions, respectively. An analysis of the regenerated spikes showed that TWG and GNPS on the regenerated spikes of wheat in the different treatments gradually decreased with an increase in stress intensity. When the temperature decreased to T4 and T5, the number of grains on the regenerated spikes in the NI condition was less than in the IBLT condition, and the number of grains on the regenerated spikes in the semi-spring cultivars was less than in the semi-winter cultivars.

### 3.4. Relationship Between Grain Yield and Yield Components Under Irrigation and Low-Temperature Stress

The multiple linear regression method was used to analyze further the relationship between single plant yield and the yield components for different irrigation conditions and temperature treatments (Table 3). The results showed that except for insignificant differences in the TWG in the T1 treatments, the SNPP, GNPS, and TWG in the other temperature treatments had a significant impact on the yield per plant; however, in the different temperature treatments and irrigation conditions, the relative variation by which each yield component influenced the yield differed. Based on the partial-R^2^ of each treatment in the NI condition, 77% and 91% of the yield variation per plant in the T1 and T2 treatments were caused by changes in GNPS, and 92%, 94%, and 93% of the yield variation per plant in the T3, T4, and T5 treatments were caused by changes in SNPP. In the IBLT-T1, IBLT-T2, and IBLT-T3 treatments, GNPS led to large variations in yield and explained 42%, 82%, and 96% of the variation in the yield per plant, respectively. In the T4 and T5 treatments, 86% and 79% of the variation in the yield per plant was caused by changes in SPNN.

Based on the standardized partial regression coefficients (SPRC), when wheat was exposed to the T1 and T2 temperature treatments, the relative contribution of GNPS was the largest under both irrigation conditions. As the low-temperature stress increased in severity, the relative contribution from GNPS began to decrease, while the contribution from SNPP increased gradually. In the T3 treatment, SNPP in the NI condition had the greatest impact on the yield per plant, mainly due to the large reduction in the number of original spikes; however, GNPS had the greatest impact on the yield per plant in the IBLT condition. In the T4 and T5 treatments at temperatures of −8 °C and −10 °C, the largest contribution to yield in the two irrigation conditions was SNPP. Based on the SPRC values, the weight of SNPP in the NI condition was greater than in the IBLT condition, indicating that at these temperatures, NI led to the death of the original tillers and the regenerated tillers produced spikes, resulting in a compensation effect on yield. In addition, more intense temperature stress results in more regenerated spikes and a greater compensatory effect.

### 3.5. Effects of Irrigation Before Low-Temperature Exposure on the Spatial Distribution of Grains per Spike in Wheat Under Low-Temperature Stress

**Figure 3:**
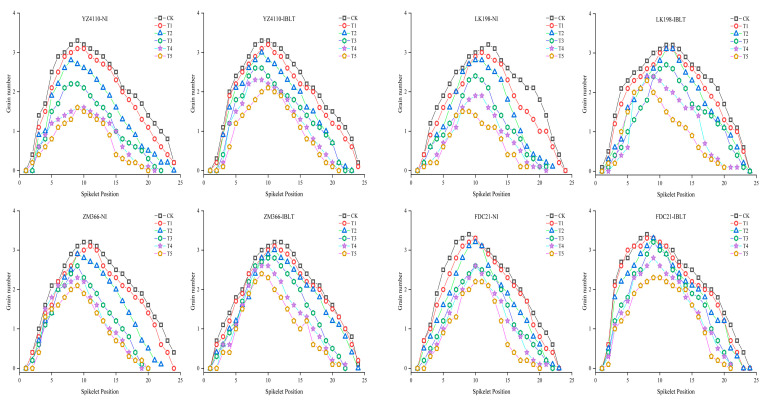
Effects of five low-temperature treatments on the spatial variation of grain number at different spike positions.

Figure 3 shows the spatial distribution characteristics of grain number per spike (when there were only regenerated spikes, we used the grain number analysis for the regenerated spikes) across four wheat cultivars exposed to varying irrigation conditions and temperature treatments. Notably, the grain number per spike initially increased and then decreased from the basal to the central region of the spike, with a higher grain count in the middle compared to the basal and the central sections. Under intensified low-temperature stress, the grain number reduced across all spike positions, with cultivar and water treatment conditions (NI or IBLT) significantly affecting the relative sensitivity to temperature. By analyzing the average number of grains (averaging the number of grains over the different spike positions), we found that for the NI condition, the grain numbers in the semi-spring cultivars (YZ4110 and LK198) continued to decrease at the lower temperatures, decreasing by 14.12% to 62.55% compared with the CK, and grain numbers in the semi-winter cultivars (ZM366 and FDC21) decreased by 10% to 50.86% compared with the CK. Under the IBLT condition, the average decrease in the number of grains per cultivar was lower than for the NI condition; the semi-spring cultivars decreased by 9.62% to 49.64%, and the semi-winter cultivars decreased by 6% to 42.57%. These results show that for the same water conditions, the average grain number reduction for the whole spike in the semi-spring cultivars was higher than in the semi-winter cultivars, while for the same cultivars, irrigation effectively reduced the degree of freezing of the young spikes, and the reduction in the average grain number in the different low temperature treatments was lower than in the no irrigated treatments.

**Figure 4:**
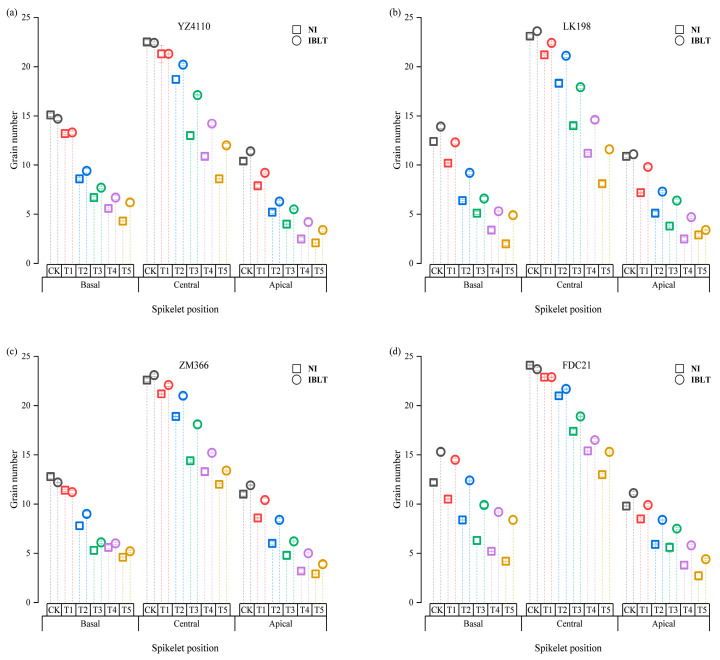
Spatial distribution of grains at different spike positions under different low-temperature stress conditions. Vertical bars represent the standard deviations. Semi-spring cultivars YZ4110 (**a**) and LK198 (**b**), and semi-winter cultivars ZM366 (**c**) and FDC21 (**d**). Symbol colors correspond to different treatments: Gray for CK, Red for T1, Blue for T2, Green for T3, Purple for T4, and Yellow for T5.

Based on Figure 4, the spatial distribution of grains at different spike positions exhibited a distinct trend as the temperature decreased. Specifically, the grain number at the apical position was most sensitive to low temperatures, followed by the basal and central spikelets. However, compared with the CK, the grains at different spike positions in the various cultivars showed different relative decreases that depended on the cultivar and irrigation condition. As the temperature decreased from T1 to T5, the number of grains in the semi-spring cultivars in the apical, central, and basal spikelets in the NI condition decreased by 29–76.6%, 6.78–63.36%, and 15.16–77.7%, respectively. In contrast, the number of grains in the spikelet from the three parts of the spike in the semi-winter cultivars decreased by 17.54–73.04%, 5.59–46.48%, and 12.11–64.82%, respectively. In the IBLT condition, the number of grains at different spike positions in each cultivar was significantly higher than in the NI condition. Compared with the CK, the number of grains in each part of the spike was reduced by 15.51–69.77%, 7.3–54.34%, and 8.21–55.59%, respectively, in the semi-spring cultivars and by 11.71–63.79%, 3.85–38.72%, and 6.71–51.24% in the semi-winter cultivars. It can be seen that the number of grains in the apical spikelet was more sensitive to low temperature, followed by the basal and then the central spikelets. The decline in grain numbers in the semi-winter cultivars at the three spike positions was less than in the semi-spring cultivars. In comparison, the declines in each cultivar in the irrigated treatments at the three spike positions were less than in the NI treatments.

**Figure 5:**
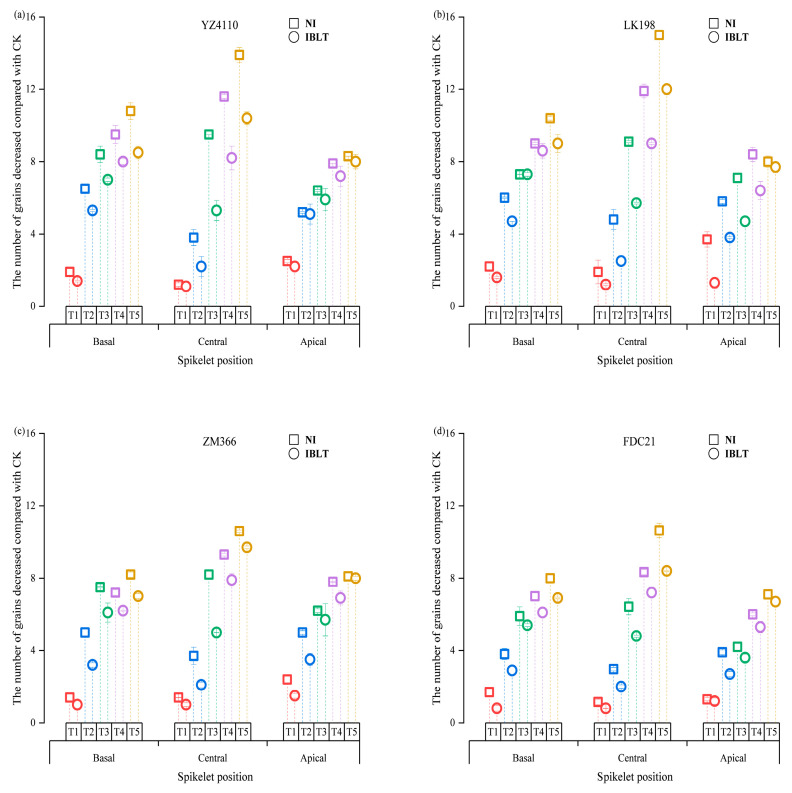
Reduction in grain numbers at different spike positions in the five low-temperature treatments ((**a**,**b**,**c**,**d**) were YZ4110, LK198, ZM366, and FDC21 respectively). Vertical bars represent the standard deviations. Cultivar names and irrigation conditions are provided in Figure 4. Symbol colors correspond to different treatments: Symbol colors correspond to different treatments: Red for T1, Blue for T2, Green for T3, Purple for T4, and Yellow for T5.

Figure 5 shows that when the low temperatures were T1 and T2, in the NI condition, the number of grains (average value) at the apical of the spike in the semi-spring cultivars and the semi-winter cultivars was reduced the most, by 3.1–6.5 grains and 1.9–4.5 grains, respectively, compared with the CK. In contrast, the number of grains was reduced by 1.75–5 and 1.35–3.1 in the two cultivar types in the IBLT condition. When the low temperatures were T3 to T5, the degree of stress increased, and the reduction in the number of grains in the central spike in the semi-spring cultivars and semi-winter cultivars increased. The reduction was higher compared with those at the upper and lower spike positions. In the NI condition, the reduction was 9.29–14.45 grains and 7.45–10.85 grains in the semi-spring and semi-winter cultivars, respectively, while the reduction in the number of grains was 5.51–11.2 and 4.90–9.05 in the IBLT condition. It can be seen that as the temperature continued to decrease, the number of grains in the apical spikelet was the most sensitive to low temperature, and the decrease in grain number was greater than in the other parts of the spike; however, as the stress intensity increased, the freezing of the central spikelet increased, resulting in larger decreases in the number of grains in the central part of the spike. Under the same irrigation condition, the grain reduction in the semi-spring cultivars was higher than in the semi-winter cultivars. For each cultivar, the reduction in the number of grains in the NI condition was higher than in the IBLT condition. The above results showed that with the increase of low temperature stress, the degree of freezing in the central of young spikelets increased, resulting in the decrease of grain number in the central far more than the basal and apical, which was also the main reason for the significant yield reduction.

### 3.6. Interactions Between Irrigation and Low Temperatures on the Physiological Parameters of Wheat Plants

Table 4 reveals that all physiological traits demonstrated significant differences in the one-way analysis. Notably, in this analysis, the F-values for each physiological trait under temperature (Tem) treatments were higher than those of the other two factors, with irrigation (Irr) following closely. In the two-factor analysis, all physiological traits were significant or highly significant under the combined effects of Irr × Tem and Cul × Tem. The Irr × Tem interaction showed the greatest effect, with F-values of all traits, except for SOD, exceeding those in the other two-factor treatments.

In conclusion, the interactions between temperature (Tem) and irrigation (Irr) play a pivotal role in driving the variation in physiological traits. Furthermore, this finding highlights the significant impact of irrigation on mitigating or exacerbating freezing damage in wheat exposed to low-temperature stress.

### 3.7. Effects of Irrigation and Low-Temperature Stress on the Physiological Indexes of Wheat Leaves

**Figure 6:**
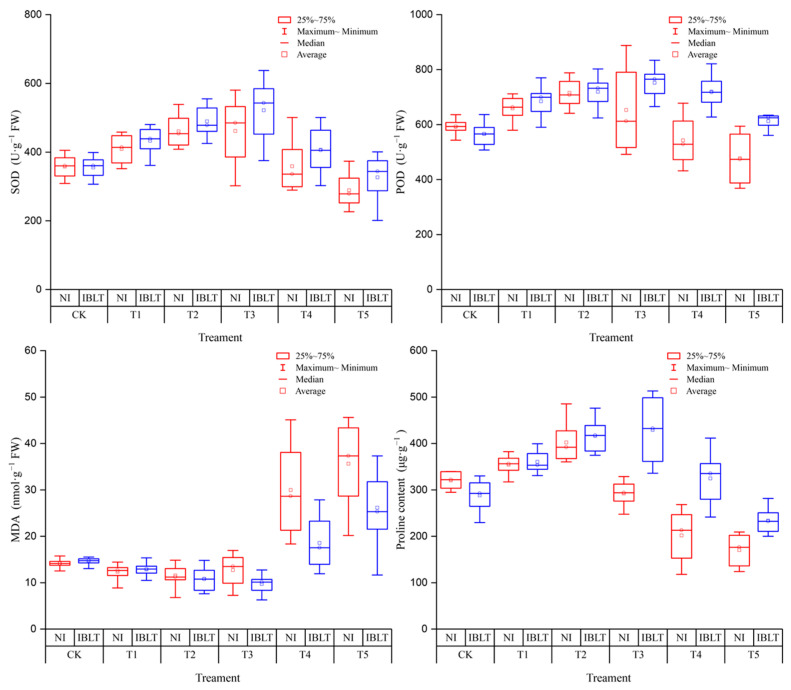
Effects of irrigation and low-temperature interactions on SOD, POD, MDA, and Pro. Symbol colors correspond to different treatments: Red for NI, Blue for IBLT.

Figure 6 illustrates the values of physiological changes for all wheat varieties in each temperature treatment and shows that as the temperature decreases gradually, the enzyme activities of SOD and POD, when exposed to NI and IBLT conditions, exhibit an initial increase followed by a subsequent decrease. However, a difference in the magnitude of change between NI and IBLT is apparent. Under the NI condition, SOD enzyme activity peaked at T2 with a significant increase of 28.75% compared with the CK, subsequently declining as the temperature decreased from T3 to T5. However, under IBLT, SOD activity reached a maximum at T3 with a remarkable increase of 46.81%. Regarding POD enzyme activity, it reached a peak under the NI-T2 and IBLT-T3 treatments, showing increases of 20.89% and 32.98%, respectively. As the stress level increased, the MDA content demonstrated an initial decrease followed by an increasing trend, reaching its lowest levels in the NI-T2 and IBLT-T3 treatments with decreases of 18.43% and 33.72% compared with the CK. The MDA content in the leaves gradually increases to a peak as the temperature decreases, regardless of whether they are subjected to IBLT or NI. Pro functions as an osmoregulatory substance; therefore, as the temperature gradually decreases, the Pro content reaches a maximum at NI-T2 and IBLT-T3, respectively, before subsequently declining. The increase in Pro content is significant, with increases of 25.60% and 49.09% compared with the CK. Similarly to the observed changes in enzyme activity, as the low-temperature stress intensifies and exceeds plant tolerance levels, a decrease in Pro content occurs.

**Figure 7:**
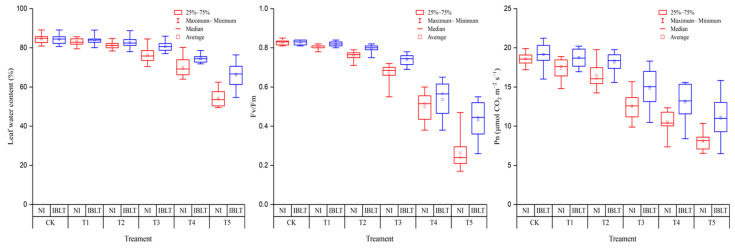
Effects of irrigation and low-temperature interactions on LWC, Fv/Fm, and Pn. Symbol colors correspond to different treatments: Red for NI, Blue for IBLT.

As the low-temperature stress intensified, the LWC, Fv/Fm, and Pn exhibited a declining trend, reaching their lowest points at the T5 temperature (Figure 7). At the T1–T2 temperature levels, reductions in the LWC when exposed to either IBLT or NI conditions were minor and insignificant. However, at T3, the decrease in the LWC became more pronounced, with an average reduction of 9.64% for NI-T3 and 4.35% for IBLT-T3 across the four cultivars compared with the CK. As temperatures decreased further to T4 and T5, the decline in the LWC continued to increase with greater reductions observed under NI than IBLT conditions; specifically, decreases of 17.08% and 35.81% for NI versus CK were observed, while those for IBLT were 11.76% and 21.69%.

The trends in Fv/Fm and Pn changes exhibited a similar pattern to that of the LWC. When the temperature decreased to T2, the decrease in Fv/Fm when exposed to NI and IBLT conditions treatments was 8.06% and 3.83% compared with the CK, respectively, while the reduction in Pn reached 11.33% and 5.24%, respectively. However, as the temperature level ranged from T3–T5, both Fv/Fm and Pn displayed an increasing decline. Under NI and IBLT condition treatments, the Fv/Fm decreased by 18.95~68.15% and 10.67~47.83%, compared with CK, respectively; meanwhile, the Pn decreased by 32.41~56.58% and 22.77~41.85%, respectively.

**Figure 8:**
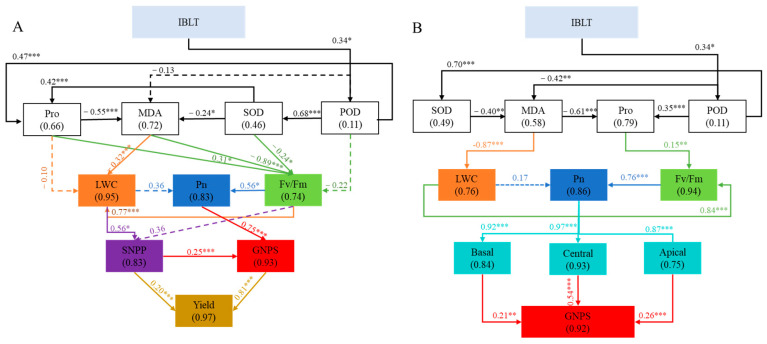
Structural equation modeling (SEM) of direct or indirect effects of physiological traits on the low-temperature response and stress capacity on Yield (**A**) and GNPS (**B**) under different moisture and low-temperature levels. ((**A**) CMIN/DF = 1.077, GFI = 0.855, RMSEA = 0.040. (**B**) CMIN/DF = 1.097, GFI = 0.891, RMSEA = 0.045). Boxes represent physiological indices names, and numbers in parentheses show the variance explained by this model (R^2^). The solid line indicates a significant positive relationship, and the dashed line indicates negative correlation. Numbers on arrows are standardized path coefficients and indicate the effect size of the relationship. A line with arrowhead indicates a putative causal link between the cause (base of the arrow) and effect (tip of the arrow). * *p* < 0.05; ** *p* < 0.01, *** *p* < 0.001.

The SEM model, integrating seven physiological indicators with SNPP, GNPS, and Yield (Figure 8A), was constructed to further explore how IBLT influenced yield through changes in physiological indicators. The results showed that IBLT had a significant positive effect on POD (path coefficient of 0.34) which played a key intermediate role in the model. And POD had highly significant positive effects on SOD and Pro (path coefficients of 0.68 and 0.42, respectively), which negatively regulated MDA (path coefficients of −0.55 *** and −0.24 *, respectively). While MDA had a significant negative regulation for LWC. Additionally, Fv/Fm was affected by Pro, MDA and SOD, all of which reached significant levels, and Pn was significantly affected by Fv/Fm. The results revealed that IBLT enhanced the plant’s stress response capability. LWC had a significant positive effect on SNPP, while Pn had a highly significant positive effect on GNPS, indicating that leaf water status and photosynthesis moderated yield. Yield was directly and positively influenced by SNPP and GNPS (path coefficients of 0.20 *** and 0.81 ***, respectively). The higher path coefficient of GNPS indicated that it was the main factor influencing wheat yield.

Figure 8B shows that IBLT promotes GNPS formation by altering physiological indicators to influence seed number in different parts of the young spike. IBLT impacted POD and SOD activities directly or indirectly. Changes in these enzyme activities directly and negatively influenced MDA levels (R^2^ = 0.58). Additionally, the extent of membrane lipid peroxidation directly and negatively impacted the LWC (R^2^ = 0.76). Osmoregulatory and antioxidant functions complement each other; hence, irrigation not only improves antioxidant capacity but also indirectly influences Pro levels. LWC and Pro content are indicative of cell structure stabilization, which positively influences the Fv/Fm (R^2^ = 0.94), with LWC having a more substantial positive effect (path coefficient 0.84). Fv/Fm directly influences the Pn (R^2^ = 0.86), which significantly and positively affects grain numbers at different spike positions. This is particularly true for the central spike, with a path coefficient of 0.97 and an R^2^ = 0.93. For GNPS, the grain number in the central part of the spike has the most positive and largest impact, followed by the grain numbers in the apical and basal parts. From the above analyses, it can be concluded that IBLT effectively mitigated the reduction in SNPP and GNPS, as well as the loss of mid-grain number, by increasing the plant’s stress and responsiveness to low temperatures, thereby reducing the cellular damage caused by low temperatures and promoting photosynthesis and stabilization of leaf water status, which ultimately mitigated yield loss.

## 4. Discussion

### 4.1. Mitigating Effect on Yield Components Losses by Irrigation Before Low-Temperature

In the context of global warming, the frequency of low-temperature events, which constitute a significant abiotic stress factor impeding wheat growth and development, has increased [43], resulting in pronounced adverse effects on wheat yield [44]. Implementing effective cultivation strategies to mitigate wheat yield losses due to low-temperature stress is essential. Snyder et al. [45] revealed that applying irrigation before the onset of frost stress enhances solar radiation absorption by the soil, consequently elevating both soil and canopy temperatures and effectively fortifying crop resistance against low-temperature stress. Wang et al. [46] identified a close association between spring frost damage to winter wheat and environmental factors, such as precipitation and soil moisture content, highlighting that judicious irrigation can effectively mitigate the impact of spring frost on wheat. Wang et al. [47] demonstrated that early irrigation can effectively reduce frost damage and yield losses of wheat. However, limited research has explored how IBLT practices affect yield and its components at different low-temperature levels. The results of this study align with prior research studies, demonstrating that the implementation of IBLT treatments led to higher wheat yields compared with NI treatments, with average yield reductions of 44.01% for NI and 33.06% for IBLT under low-temperature conditions, and notably, 43.12% (NI) and 29.35% (IBLT) under T3 (−6 °C) conditions. The yield component analysis revealed significant differences in SNPP, TGW, and GNPS between treatments, with reductions of 34.99%, 6.70%, and 24.93% for SNPP, TGW, and GNPS under NI, in contrast with 16.39%, 2.57%, and 15.44% in IBLT. The application of irrigation was identified as an effective measure to alleviate low-temperature stress by enhancing soil moisture content, insulation, and cold resistance, consequently mitigating primary tiller mortality and diminishing declines in yield components. In NI-T3, the increased mortality of primary tillers due to low temperatures resulted in a more pronounced reduction in SNPP compared with IBLT-T3, while IBLT exhibited significant tiller reduction at T4. Furthermore, semi-spring varieties exhibited a more significant reduction in yield components (SNPP, GNPS, TGW) compared with semi-winter varieties, with differential yield reduction patterns observed in semi-spring (YZ4110, LK198) and semi-winter (ZM366, FDC21) varieties. Semi-spring varieties experienced average reductions of 47.69% and 36.14% under NI and IBLT conditions, while semi-winter varieties showed reductions of 40.32% and 29.98%, respectively. The collective findings indicated that strategically selecting cold-resistant varieties, combined with implementing irrigation management before frost onset, represents an effective approach to mitigate yield losses.

Wu et al. [19] found a correlation between producing regenerative tillers in wheat and frost damage severity, where severe frost damage results in the freezing death of young spikes on the main stem, impeding regenerative tiller formation. Furthermore, temperatures that are insufficiently or excessively low are ineffective in promoting regenerative tiller production. In this investigation, the compensatory effect on yield observed in the NI-T3 and IBLT-T4 treatments is attributable to the redistribution of nutrients induced by primary tiller death, promoting the rapid growth of new tillers from axillary buds and resulting in regenerative spike development [20,48]. In the IBLT condition, wheat presented a lower regenerated spike count across all temperature levels compared with the NI treatment, indicating that irrigation mitigates frost damage, promotes primary tiller survival, and consequently diminishes the number of regenerated spikes. Moreover, regenerated spikes, characterized by a lower 1000-grain weight (R-TGW) and number of grains per spike (R-GNPS), exhibited reduced values compared with the original spikes (O-TGW and O-GNPS), attributable to delayed development and inadequate nutrient supply, consequently leading to diminished GNPS and TGW. Thus, although regenerated spike yield provides partial compensation for overall yield loss, it fails to completely offset the adverse impact of low temperatures on primary spike yield.

### 4.2. The Contribution of Yield Components to Yield and Changes in Grain Distribution at Different Spikelet Positions Under Low Temperatures

The primary cause of yield loss due to low temperatures lies in the reduction of both spike number and length [49]. Indirect causes include the effects of low temperatures on the differentiation of young spikelets [50], a reduction in photosynthetic capacity [14], and consequently, a decrease in dry matter accumulation and grain filling rate [35]. These changes ultimately lead to a reduction in GNPS and TWG. A previous study showed that SNPP and GNPS were the most significant factors affecting yield variability under low-temperature stress, contributing more overall than SNPP and TWG [21]. In this study, we examined the sensitivity of SNPP, GNPS, and TGW to low-temperature stress under various irrigation treatments, assessing their contributions to yield using the standardized regression coefficient method. SNPP is associated with the number of effective tillers, whereas GNPS correlates with young spike development. A significant reduction in the number of effective tillers per plant, induced by low-temperature stress at the jointing stage, resulted in notable declines in both SNPP and GNPS, establishing them as predominant factors contributing to yield reduction. As the temperature decreased, the primary factor influencing yield variation transitioned from GNPS to SNPP upon reaching specific temperature thresholds, namely T3 (−6 °C) in NI and T4 (−8 °C) in IBLT. Simultaneously, the results suggest that mild to moderate stress conditions (T1–T2) detrimentally impacted the development of young spikes, resulting in heightened sterility of florets within young spikelets and a diminished grain fertility rate, which have a more significant influence on yield than both the GNPS and the TGW. Under severe stress conditions (T3–T5), low-temperature stress exceeds the plant’s tolerance capacity, leading to a reduction in the number of effective tillers and the death of young spikelets. This means that the extent of severe stress’s impact on yield is caused by a reduced number of effective tillers and the death of young spikelets, which is more substantial than the influence of GNPS.

GNPS is a critical determinant of yield and represents a significant constraint on enhancing wheat yield [51]. In the development of young spikelets, floret development plays a pivotal role in determining the final grain count. Fertile floret development, which directly influences the GNPS, is influenced by factors such as light availability, growth of nutritive organs, and availability of nutrients and water, along with external environmental factors [27,51,52,53]. Considering the varying sensitivities of spikelets and grains at different spike positions to low temperatures, elucidating the influence of irrigation on the spatial distribution of grain numbers in spikelets under different levels of low-temperature stress is imperative. Liu et al. [20] demonstrated that in young wheat spikelets, floret differentiation prioritizes the central spikelet, followed by the apical and basal positions. During the jointing stage, the apical and basal florets of young spikelets are highly sensitive to low temperatures, rendering them prone to injury, which in turn leads to floret abortion and subsequent reductions in grain yield. The findings of this study align with those of previous research, indicating that spikelets at the apical position are the most sensitive to low temperatures, followed by those at the basal and central positions. Additionally, a pronounced decrease in grain number when exposed to low-temperature stress is notably more significant in the apical spikelet compared with those at the basal and central positions, with semi-spring wheat varieties exhibiting more substantial grain losses compared with semi-winter varieties across diverse spikelet numbers. Furthermore, semi-spring and semi-winter varieties exhibited reduced grain losses at different spikelet positions under IBLT and compared with the NI CK. The grain loss in the IBLT condition ranged from 13.61% to 66.78% at the top, 5.58% to 46.53% in the middle, and 7.46% to 53.42% at the base, in contrast with 23.27% to 74.82%, 6.19% to 54.92%, and 13.64% to 71.26% under NI conditions. The distribution of grain numbers in wheat spikelets exhibited a near–median dominance pattern, where the central spikelet typically had the highest proportion of grains, surpassing both the basal and apical spikelet [50]. This study unveiled that, at lower temperature levels (T1–T2), the most substantial reduction in grain number was observed in the apical and basal positions in the spike; as temperatures declined to T3 and below, the decrease in grain numbers at the central spike position progressively exceeded that at the apical and basal positions. Particularly, under intensifying stress, this reduction in the central position became more pronounced. Analyzing the extent of grain reduction in the central spike position, it became apparent that semi-spring varieties exhibited a more substantial decrease than semi-winter varieties, with the reduction being notably higher in the NI treatments compared with the IBLT treatments. In summary, this study found that under low-temperature conditions, the reduction in grain numbers at different spike positions varied depending on the variety and irrigation conditions. As the temperature decreased further, the initial reduction in grain numbers primarily began with a decrease in the apical and basal grains. When temperatures reached T3 (NI) and T4 (IBLT), the reduction in grain numbers at the central spike position escalated, outstripping those at the basal and apical positions. This cumulative reduction in grain numbers across the apical, central, and basal spike positions resulted in a significant overall decrease in the GNPS, leading to a substantial reduction in yield. Moreover, the application of IBLT significantly alleviated the reduction in grain numbers at diverse spike positions, providing valuable scientific insights and technical support for addressing low-temperature stress during the jointing stage in agricultural practices.

### 4.3. Physiological Mechanism by Which Irrigation Alleviates the Effects of Low Temperature on Wheat Grains

As low-temperature levels continue to decrease, the leaves gradually yellow and wilt, and young spikelets exhibit stunted growth. With intensifying stress, dehydration occurs, ultimately leading to plant death. Importantly, cell membranes demonstrate heightened sensitivity to low-temperature stress, with physiological responses manifesting earlier than visible morphological changes. Stress induces the activation of plant antioxidant enzymes, accumulation of osmoregulatory substances, scavenging of excess ROS, and maintenance of intracellular osmotic balance, effectively mitigating the damage incurred by cell membranes [27,28,54]. However, when the stress level exceeds the frost tolerance limit of plants, irreversible damage ensues, as ROS production outpaces the scavenging capacity of antioxidant enzymes, leading to elevated MDA levels, reduced antioxidant enzyme activity, disrupted cell membranes, osmoregulatory imbalances, and a decline in associated traits, ultimately culminating in cell death. The disruption of cell membranes leads to reduced LWC, chlorophyll degradation, harm to photosynthetic organs, irreversible changes in fluorescence parameters, and a subsequent decrease in photosynthetic capacity [23,55,56]. Most of these studies focused on the effects of low-temperature stress on plant physiological traits, but few on the interactions of irrigation and low temperature on plant physiological traits. This study unveiled that the interaction between moisture and temperature exerted a paramount influence on physiological traits, with the IBLT condition showcasing heightened responses in the antioxidant and osmoregulation systems at lower temperatures (IBLT-T3), particularly characterized by increased SOD and POD enzyme activities, along with elevated Pro content. Conversely, in the NI-T3 treatment, the cell membranes exhibited signs of damage, with reduced enzyme activities and Pro content compared with NI-T2. Additionally, LWC, Pn, and Fv/Fm experienced significant declines under NI-T3 and IBLT-T4 conditions, indicating substantial impacts on both the moisture effects and photosynthetic effects of plants exposed to severe low-temperature stress.

Photosynthesis, a fundamental process for energy conversion and carbon metabolism in plants, is significantly disrupted by low-temperature stress across various growth stages, resulting in decreased photo-assimilation and assimilate transportation, ultimately leading to significant yield loss [24,35,38]. Measures such as fertilization and irrigation have been shown to compensate for these losses [27,28,35]. Although the current literature suggests that irrigation can alleviate wheat yield reductions induced by low temperatures [37,47], most studies focus on soil moisture level impacts on yield. To explore the influence of irrigation on grain number formation through its effects on physiological characteristics, an SEM model was constructed. The results revealed that irrigation inhibited oxidative bursts in the photosynthetic system, lowered cellular freezing points by enhancing antioxidant enzyme activities, and increased levels of osmoregulatory substances. Moreover, irrigation maintained cellular expansion by sustaining high leaf water potential, elevating the photosynthetic rate, and mitigating the impact of low-temperature stress on wheat leaf photosynthesis. This role of LWC as a significant positive influence on SNPP and Pn for GNPS was evident from Figure 8A. The higher the leaf water potential, the greater the resistance of young spikelets to dehydration [36]. Higher water potential also improves the development of young spikelets by increasing photosynthesis, and in turn increases the final yield. According to the SEM model (Figure 8B), three portions of the young spikelet were markedly positively influenced by photosynthesis, with the highest path coefficient observed in the central spikelet, followed by the basal and apical sections. This suggests that, when damage occurs to different spikelet sections, there is a preferential allocation of photosynthetic products and nutrients to the less affected regions. This adaptive allocation supports the development of central and basal florets, ultimately increasing grain count per spikelet and reducing the adverse impact of floret failures induced by low temperatures on overall yield.

## 5. Conclusions

This study highlights the significant interaction between IBLT and low-temperature stress, which notably impacts wheat yield and its components. Yield, SNPP, GNPS, and TWG experienced mitigated reductions under IBLT compared to NI conditions, with SNPP and GNPS emerging as pivotal factors influencing yields during low-temperature stress. Moreover, when stress exceeds the plant’s tolerance (as in NI-T3 and IBLT-T4 conditions), the determining factor of yield shifts from GNPS to SNPP. Low temperatures impacted spikelet positions differently, with apical spikelets being the most sensitive, followed by the basal and central ones. IBLT enhanced cold tolerance by boosting antioxidant enzyme activities and osmoregulatory substances, mitigating damage to the photosynthetic system and preserving leaf water potential, thereby ensuring greater availability of photosynthetic products for the growth of young spikelets. Photosynthesis positively impacted each portion of the young spikelet, with the central section deriving the greatest benefit. In conclusion, in regions susceptible to spring frosts, selecting semi-winter wheat varieties with robust cold tolerance and implementing IBLT in response to weather fluctuations are essential for enhancing cold protection in wheat fields and minimizing frost damage impacts.

## Figures and Tables

**Table 1:** Characterization of wheat cultivars used in the experiment.

Cultivar	Breeding Unit	Certification Unit	Information
Yanzhan4110	Henan Yuxi crop variety Exhibition center, Luoyang, China	China National Crop Variety Examination and Approving Committee, 2003032	Semi-spring cultivar
Lankao198	Henan Tianmin seed industry Co., Ltd., Kaifeng, China	China National Crop Variety Examination and Approving Committee, 2014009	Semi-spring cultivar
Zhengmai366	Wheat Research Institute, Henan Academy of Agricultural Sciences, Zhengzhou, China	China National Crop Variety Examination and Approving Committee, 2005003	Semi-winter cultivar
Fengdecun21	Henan Fengdekang seed industry Co., Ltd., Zhengzhou, China	China National Crop Variety Examination and Approving Committee, 20210051	Semi-winter cultivar

**Table 2:** Effects of irrigation before low-temperature exposure on grain yield and yield components for the original (O) and regenerated (R) spikes in four wheat cultivars.

		YZ4110				LK198				ZM366				FDC21			
		IBLT		NI		IBLT		NI		IBLT		NI		IBLT		NI	
		O	R	O	R	O	R	O	R	O	R	O	R	O	R	O	R
SNPP	CK	3.3 a	-	3.28 a	-	3.39 a	-	3.33 a	-	3.57 a	-	3.45 a	-	3.46 a	-	3.42 a	-
	T1	3.27 a	-	3.21 b	-	3.37 a	-	3.27 b	-	3.52 a	-	3.38 b	-	3.45 a	-	3.35 b	-
	T2	3.05 c	-	2.82 d	-	2.98 c	-	2.79 d	-	3.35 b	-	3.13 c	-	3.31 b	-	3.11 c	-
	T3	2.61 e	-	1.92 f	1.28 d	2.65 e	-	1.64 f	1.33 d	3 d	-	2.51 e	0.5 e	2.92 d	-	2.72 e	0.4 c
	T4	1.5 g	1.61 c	-	2.35 b	1.53 i	1.4 c	-	2.51 a	1.77 f	1.09 d	1.17 g	1.47 b	1.91 f	1.25 b	1.38 g	1.63 a
	T5	-	2.6 a	-	1.58 c	-	1.95 b	-	1.14 e	1.21 g	1.3 c	-	1.76 a	1.25 h	1.15 b	-	1.67 a
TWG	CK	45.61 a	-	45.18 a	-	45.06 a	-	43.46 ab	-	45.9 a	-	45.82 a	-	46.93 b	-	46.5 b	-
	T1	45.38 ab	-	44.28 b	-	44.91 a	-	41.44 c	-	45.8 a	-	44.89 b	-	46.56 b	-	45.67 c	-
	T2	43.25 c	-	42.6 d	-	42.6 c	-	40.11 d	-	43.91 b	-	43.4 bc	-	45.16 d	-	44.37 e	-
	T3	44.8 ab	-	40.65 e	37.83 a	43.33 bc	-	38.61 e	34.34 b	44.21 a	-	41.84 c	36.26 a	46.47 a	-	43.32 f	37.26 a
	T4	39.99 e	37.95 a	-	34.08 c	39.02 e	36.78 a	-	32.29 c	41.88 c	35.84 a	44.92 ab	34.55 a	43.28 f	36.5 a	43.99 ef	35.26 b
	T5	-	35.65 b	-	32.04 d	-	29.07 d	-	31.26 c	39.59 d	31.81 b	-	34.75 a	40.71 g	37.47 a	-	33.91 c
GNPS	CK	43.5 a	-	42.65 ab	-	48.8 a	-	46.84 a	-	43.1 a	-	42.6 a	-	46.5 a	-	45.35 a	-
	T1	42.4 b	-	40.8 c	-	47.1 b	-	45.29 c	-	42.7 a	-	41.5 b	-	46 a	-	44.9 b	-
	T2	40.05 c	-	38.6 d	-	45.51 c	-	42.8 d	-	40.5 b	-	39.2 c	-	43.55 c	-	41.99 d	-
	T3	36.5 e	-	27.45 g	26.1 b	40.85 e	-	32 f	25.6 a	37.7 d	-	31.2 f	26.75 b	39.75 e	-	36.2 g	31.8 b
	T4	28.95 f	27.35 a	-	23.1 c	30.05 g	25.9 a	-	22.3 b	33.1 e	28.25 a	25.9 h	24.15 c	35.3 f	36.15 a	29.35 h	30.65 c
	T5	-	25.35 b	-	19.9 d	-	23.85 b	-	18.9 c	28.5 g	26.85 b	-	22.75 d	32.25 h	26.1 d	-	24.55 e

Notes: SNPP, GNPS, and TGW indicate spike number per plant, grain number per spike, and 1000-grain weight. Vertical bars represent the standard deviations. Values for the same wheat cultivar type followed by different lowercase letters indicate significant differences at 0.05 level.

**Table 3:** Relationships between yield and components in wheat exposed to different low-temperature and irrigation treatments.

Irrigation Condition	Yield Component	T1		T2		T3		T4		T5	
		SPRC	Partial R^2^	SPRC	Partial R^2^	SPRC	Partial R^2^	SPRC	Partial R^2^	SPRC	Partial R^2^
NI	SNPP	0.35 *	0.52	0.57 **	0.91	0.66 **	0.92	0.85 **	0.94	0.81 **	0.93
	GNPS	0.66 **	0.77	0.63 **	0.95	0.58 **	0.84	0.51 **	0.93	0.52 **	0.87
	TWG	0.34 *	0.51	0.16 *	0.50	0.45 **	0.81	0.29 **	0.71	0.38 **	0.80
IBLT	SNPP	0.47 **	0.42	0.6 **	0.81	0.37 **	0.89	0.49 **	0.86	0.62 **	0.79
	GNPS	0.49 **	0.42	0.61 **	0.82	0.64 **	0.96	0.42 **	0.85	0.27 **	0.54
	TWG	0.28	0.27	0.28 *	0.54	0.54 **	0.95	0.46 **	0.84	0.24 **	0.57

*: *p* < 0.05; **: *p* < 0.01. SPRC, standardized partial regression coefficient.

**Table 4:** Variance analysis of physiological and biochemical indexes and chlorophyll fluorescence parameters.

Treatment	SOD	POD	MDA	Pro	LWC	Fv/Fm	Pn
Irr	169.91 ***	247.85 ***	79.95 ***	336.55 ***	69.39 ***	88.82 ***	75.59 ***
Cul	471.59 ***	167.41 ***	41.36 ***	115.05 ***	10.79 ***	32.47 ***	24.99 ***
Tem	528.44 ***	164.89 ***	232.31 ***	472.23 ***	270.66 ***	737.3 ***	190.46 ***
Irr × Cul	11.2 ***	21.62 ***	7.97 ***	3.94 *	1.63	5.18 **	1.25
Irr × Tem	12.48 ***	55.71 ***	23.51 ***	95.8 ***	14.56 ***	18.23 ***	2.66 *
Cul × Tem	39.42 ***	8.42 ***	9.51 ***	10.75 ***	3.97 ***	5.48 ***	1.89 *
Irr × Cul × Tem	4.45 ***	7.5 ***	3.42 ***	3.4 ***	0.98	2.25 **	0.65

* *p* < 0.05; ** *p* < 0.01; *** *p* < 0.001: Irr, irrigation, Cul, cultivar, Tem, temperature, SOD, Superoxide Dismutase, POD, Peroxidase, MDA, Malondialdehyde, Pro, Proline, LWC, Leaf Water Content, Fv/Fm, The ratio of variable to maximum fluorescence, Pn, Photosynthetic Rate.

## Data Availability

Data are contained within the article.
